# Primary health care coverage in Portugal: the promise of a general practitioner for all

**DOI:** 10.1186/s12960-024-00936-7

**Published:** 2024-08-09

**Authors:** Eduardo Costa, Joana Pestana, Pedro Pita Barros

**Affiliations:** 1 CEGIST - Centre for Management Studies, Instituto Superior Técnico, Universidade de Lisboa, Av. Rovisco Pais 1, 1049-001 Lisbon, Portugal; 2 Nova School of Business and Economics, NOVA University Lisboa, Campus de Carcavelos, Rua da Holanda 1, 2775-405 Carcavelos, Portugal

**Keywords:** Primary care, General practice, Patient lists, Family doctor, Portuguese NHS

## Abstract

**Background:**

Primary care is an essential pillar of health systems. Many countries have implemented different policies to improve access to primary care. However, persistent challenges remain. This paper offers a critical analysis of the evolution of primary care coverage in Portugal, focusing on the number of patients without an assigned general practitioner (GP).

**Methods:**

We collected and analyzed publicly available data from 2009 to 2023 to decompose primary care coverage in three components: the number of patients enrolled in primary care units (demand-side effect), the number of GPs measured in full-time equivalent (supply-side effect), and the average number of patients on each GP’s list (patient-to-GP ratio, capturing a productivity effect). We provide national and local level estimates for these three components.

**Results:**

Between 2009 and 2023, there was an overall decline in the number of patients enrolled in primary health care units. Concurrently, there was also a net decrease of GPs measured in full-time equivalent. Additionally, there was a progressive reduction in the average number of patients on each GP’s list. The rise in the number of patients without an assigned GP is attributed not only to a reduction in the number of physicians, but also to a decrease in the patient load per doctor.

**Conclusions:**

Hiring additional GPs may not suffice to enhance coverage. Achieving higher coverage may imply revisiting patient load per doctor or considering alternative care models. Understanding the challenges related to GP coverage is critical for improving the efficiency of primary care.

## Introduction

Currently, health systems face multiple challenges related to shortage of health professionals, increasing demand and limited budgets. An efficient primary care system is a necessary condition to deliver high-quality care [1]. However, achieving universally primary care depends not only on organizational adaptability of health systems, but also on the availability of human resources.

This paper provides a critical analysis of the evolution of primary care coverage in Portugal. We compile publicly available data to decompose primary care coverage in three effects: the variation in the number of enrolled patients in primary care (demand-side), employed physicians measured in full-time equivalent (supply-side), and physicians’ list (patient-to-GP ratio, capturing a productivity effect). We then discuss the challenges related to achieving universal coverage.

The worldwide shortage of health professionals prevents health systems from attaining universal health coverage goals. In the US, estimates point towards a deficit of 139 160 physician professionals by 2030 [2]. Similarly, a shortage of nearly 400 000 physicians and of nearly 2.5 million nurses is forecasted by 2030 in OECD countries [3]. The 2022 WHO report on health and care workforce in Europe points out that countries struggle to attract and retain health professionals [4]. Worldwide, a shortage of up to 15 million health workers is predicted by the end of the decade [5]. Furthermore, the uneven distribution of physicians across regions results in underserved areas [6].

Shortages in the healthcare workforce can be attributed to a myriad of factors, with various challenges emerging both on demand and supply sides. On the demand-side, the aging population in many regions, coupled with increasing prevalence of chronic diseases, has contributed to the increase in healthcare needs [7]. Projections for the United States indicate a need for an additional 10 000 physicians in primary care until 2025 to adequately address the challenges associated with population aging [8]. Moreover, rising population expectations for healthcare services, coupled with increased access to information and personalized treatments, have intensified the demand for health professionals [9]. The unprecedented challenges posed by the COVID-19 pandemic have only served to exacerbate the workload on these professionals.

On the supply-side, the declining attractiveness of health professions is influenced by several factors. Physicians tend to prefer urban and suburban areas, leaving rural and underserved populations with inadequate healthcare access and potentially exacerbating health inequities [6]. The insufficient investment in state-of-the-art facilities and advanced technology hampers the efficiency of healthcare services. Specifically, primary care relies on the establishment of robust patient–doctor relationships and proves to remain particularly labor-intensive despite the existence of health-related apps and technical support programs for physicians [10]. Deteriorating working conditions further contribute to shortages, as health professionals grapple with environments that may not adequately support their well-being and productivity. This ranges from stagnant remunerations in the context of escalating living costs, to rigid job positions which limit flexibility and creates a financial disincentive. This lack of flexibility can make it challenging to tailor employment contracts to health professionals’ preferences, which increasingly value autonomy [11].

In primary care, a key challenge for policy-makers is the recruitment of general practitioners (GP). The inadequacy of resources, or their maldistribution, further hinders the achievement of this goal. Some reforms have been implemented to improve the relative attractiveness of primary care [12]. These include efforts to reduce physicians' administrative workload, automate routine tasks such as health record management, and provide improved access to essential skills and support. However, many of these reforms have often fallen short of their intended objectives [13].

In recent years, the Portuguese National Health Service (NHS) has undergone multiple reforms to enhance access to primary care. The NHS provides publicly financed health care to the population, even though private health care services are also available. Over the last decades, political parties have promised to assign a GP to every resident in Portugal. This has been both a frequent electoral pledge, as well as a milestone included in multiple government programs, at least since the beginning of the century. Despite the variety of policies implemented and increased funding, the goal of a GP for all residents has not yet been achieved.

In a country with a population of around 10.3 million inhabitants, 16.5% of patients in primary care had no assigned GP in 2023. These patients have significant access constraints to primary care. For these patients, access is privileged only for acute conditions and contingent on physicians’ limited availability. The lack of access to primary care may force patients to search for private health care services or, within the NHS network, to enter hospital care through the emergency department. The proportion of patients without an assigned GP in 2023 was slightly above 2009 levels (15.8%). However, it was significantly above 2019 levels, where primary care coverage was at its maximum level—with only 7.3% of patients without an assigned GP.

A key factor that influences the number of patients without GP is related with the ability of the NHS to retain primary care doctors with attractive working conditions [14, 15]. However, such ability is challenged by many different factors. First, despite policies implemented by the NHS to attract GPs to underserved areas, evidence suggests that GPs tend to concentrate in locations with more favorable working conditions [16]. Second, the dual practice context of the Portuguese health system imposes additional nuances. In fact, in 2023, a total of 8 856 GPs were licensed to practice in Portugal, with 6 934 (78%) of those being employed in the NHS (either in full-time or part-time) [17]. Although precise data are not available, a survey revealed that two-thirds of physicians did not work full-time in the public sector, and more than half also worked in private practice [18]. Third, this situation may also be exacerbated by the impending retirements, since 1 489 GPs (17%) were aged 50–65 in 2022 [17].

Moreover, migration of physicians to other countries may impose further challenges to the Health System, including in primary care. Although no specific data exists regarding GPs, research among medical residents and junior doctors suggests that 55–65% were considering the possibility of moving abroad in the near future [19, 20]. In 2023, the Portuguese Physicians Association claimed that over 450 physicians manifested their intention to migrate (around 2 000 physicians in the period between 2019 and 2023) [21, 22]. Although this represents less than 1% of doctors registered in Portugal (63 053), it corresponds to roughly 22% of new medical residents in 2023 (2 044) [23].

The shortage of GPs in the Portuguese NHS primary care network can be proxied by the number of job openings defined by the Health Ministry, since the hiring for primary care is centralized and happens periodically, once or twice a year. In the last three hiring seasons, between 900 and 1 000 vacancies were opened [24, 25]. However, applications were substantially lower: in December 2023, there were only 114 hired physicians for a total of 924 vacant positions [25].

This is the first study that describes the primary care coverage in Portugal over more than one decade, from a pre-financial crisis and austerity measures period to a period post the COVID-19 pandemic. Some of the issues faced by the Portuguese NHS primary care workforce are shared by many other health systems, particularly in Europe. Hence, improving the discussion on their root causes and potential solutions is key to generating actionable contributions to other health systems struggling with similar challenges, as discussed by the editorial for the special collection on the medical workforce crisis in primary care in Europe [26].

## Institutional background

The Portuguese NHS, a publicly funded healthcare system, offers comprehensive and universal healthcare coverage to the population [27], although private healthcare also exists. Nonetheless, the NHS represents roughly two-thirds of overall public health expenditure [28].

Within the Portuguese NHS, primary care services are delivered through public health centers, where GPs, also known as family doctors, work in teams of professionals and serve as gatekeepers. GPs operate on a patient list basis, and while all residents have the option to enroll in a primary care unit of their catchment area, this does not guarantee assignment to a GP. Residents can opt out of the NHS primary care network and seek care in the private sector through voluntary health insurance or out-of-pocket payments. Nonetheless, almost all residents are enrolled in a primary care unit, either in a GP list or awaiting vacancy in any GP list of the practice where they are enrolled. In 2023, 10.5 million patients were enrolled in primary care units, exceeding the 10.3 inhabitants according to the 2021 census, which reflects additional patients such as migrants.

In 2023, of the enrolled patients in primary care, 16.5% were not assigned to a GP. Consequently, these patients face significant constraints in accessing the NHS—especially when considering the GP’s gatekeeping role. While they can request acute same-day appointments at their primary care units, availability is contingent on the physician's schedule, which often has limited openings.

In 2005, a primary care reform was launched with the goal of improving quality of care. Primary care practices underwent administrative consolidation with the creation of regional groups of primary care practices (ACES) [27]. Under this reform, a new organizational and financing model was also rolled out: the Family Health Units (FHU). A team of physicians, subject to prior assessment and budgetary availability, could voluntarily apply to transition their practice into a FHU unit [29]. These teams are contracted to provide care for a specific geographically defined population, with an average of 1,900 patients assigned to each physician. In these units, physicians, nurses and administrative staff receive an individual monetary incentive linked to their performance, which in the case of physicians can represent up to 60% of their salaries. A transitional model (FHU-A) with pay-for-performance incentives attributed to the team was created, for units transitioning between the traditional model (Personalized Health Care Unit, PHCU) to the full FHU model with individual incentives and more stringent performance targets (FHU-B).

Those physicians who choose not to establish or join FHU would continue to operate in practices following the prior model (PHCU). In the PHCU model, while also having a fixed list of patients, physicians are paid a fixed salary. Moreover, as FHU are being established almost exclusively with patients with a GP, patients without a GP tend to concentrate in the remaining PHCU units. This implies that physicians in PHCU not only manage patients in their list but are also responsible for providing care to patients without an assigned GP, enrolled in their practices.

The roll-out of FHU was slow and subject to budget constraints. In 2007, there were 81 FHUs (12.9% of the practices). By the end of 2023, the number of FHUs had increased to 619 (68.2%), employing 4 107 GPs (76.1%) and covering 7.2 million patients (69.5%). Among these patients, only 3.65% were not included in a GP list. In the traditional model (PHCU), 1 290 GPs (23.9%) were providing care to 30.5% of all enrolled patients, of which 40.5% were not assigned to a GP [30].

## Methods

This paper presents descriptive evidence on GP coverage for Portuguese residents, examining its evolution over time and decomposing it into supply, demand and productivity effects. Data for this study were gathered from several official sources, specifically the Access Report from the Central Administration of the Health System [31], the NHS Transparency database [14], and the primary healthcare monitoring dashboards (BI-CSP) [30].

The key variables of interest include (i) the number of enrolled patients in primary care practices (demand-side effect); (ii) the number of GPs in primary care practices measured in full-time equivalents (supply-side effect); and (iii) the number of patients without an assigned GP.

This information was collected from 2009 to 2023 for each group of primary care practices (AceS), consisting of 55 units (74 units from 2009 to 2012). Further contextual variables were also collected to conduct secondary analysis. Data were aggregated both at the level of five regional health administrations (ARS) and at the national level. Only mainland Portugal was considered.

To ensure accuracy and prevent potential double-counting of physicians working in multiple practices simultaneously, we adopted the full-time equivalent (FTE) method to calculate the weighted number of GPs. Not adjusting the number of GPs to full-time equivalent would impose a bias in our results, namely in terms of the patient-to-GP ratio. According to the data from December 2023, there were 6 934 GPs working in public primary care practices, which corresponds to 5 395 GPs FTE [30]. This means that in each ACES the number of GPs FTE corresponds to 83.9% of the total number of GPs. To calculate the FTE, the effective working hours were compared versus the working hours of the 40 h per week of the current normal working period for Specialist and Intern Physicians. Throughout the paper, mentions to the number of GPs refer to GPs measured in FTE.

The analytical approach is twofold. First, we provide an overview of GP coverage, detailing the observed trends. Second, we conduct a decomposition of GP coverage, distinguishing between demand, supply and productivity effects at the national and local level.

The calculation of the number of patients without an assigned GP (${m}_{j}$) in each group of primary care practices (AceS) (*j)* is determined by several factors. Firstly, it depends on the total number of patients enrolled at each group of primary care practices (${n}_{j}$). An increase in the number of patients enrolled increases the difficulty of providing coverage for all. Secondly, it is contingent on the number of full-time equivalent physicians working at each group of primary care practices ($\sum {GP}_{i}$). The recruitment of additional physicians enhances overall coverage. Thirdly, it relies on the number of patients each physician (*i*) has on her own list (${L}_{i}$). Larger patient lists per physician contribute to increased coverage, assuming all other variables remain constant. Therefore, the estimation of patients without an assigned GP was derived from the following expression:

1 $${m}_{j}={n}_{j}-\sum {GP}_{i}{L}_{i}.$$

This expression can be simplified considering the average number of patients with GP per doctor (patient-to-GP ratio) in each group of primary care practices (${L}_{j}$). This represents the ratio between patients with an assigned GP and the number of doctors. Thus, the previous expression can be written has a function of variables collected for each group of primary care practices *j*:

2 $${m}_{j}={n}_{j}-{GP}_{j}{L}_{j}.$$

One can use the previous expression to decompose the change in the number of patients without GP into different effects. The following expression represents the decomposition of the change on the number of patients without an assigned GP between two periods (0 and 1) in three effects—each one within brackets: change in the number of enrolled patients, change in the number of physicians, and change in the patient-to-GP ratio (the average list size per physician):

3 $${m}_{j}\left(1\right)-{m}_{j}\left(0\right)=\left[{n}_{j}\left(1\right)-{n}_{j}\left(0\right)\right]-\left[\left({GP}_{j}\left(1\right)-{GP}_{j}\left(0\right)\right){L}_{j}\left(0\right)\right]-\left[\left({L}_{j}\left(1\right)-{L}_{j}\left(0\right)\right){GP}_{j}\left(1\right)\right].$$

## Results

### Descriptive evidence

**Fig. 1:**
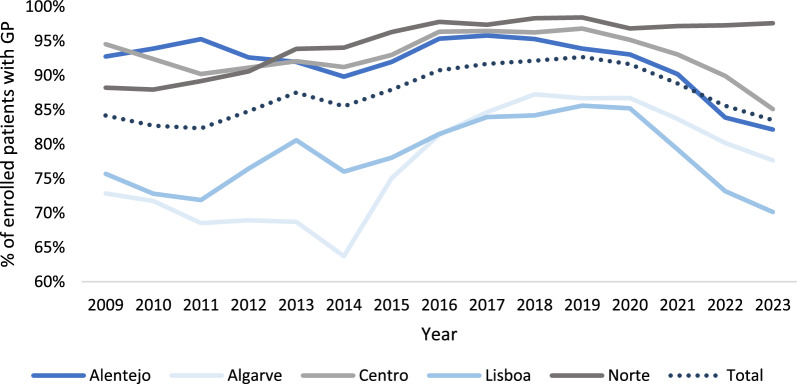
Patients enrolled in a primary care practice with an assigned GP per administrative region (% of enrolled patients in primary care units; 2009–2023)

Universal coverage of primary care, by granting a GP to each resident, has been a political objective since the onset of the NHS. Figure 1 provides the proportion of patients enrolled in primary care practices with an assigned GP between 2009 and 2023 for each of the five administrative regions.

In 2023, 83% of the population nationwide had an assigned GP. This was close to the historical minimum of 82% coverage verified in 2011, and well below the 93% maximum coverage registered in 2019. One can see that, even though sizeable differences are identified across regions, there is a common pattern among them. Nationwide, GP coverage had a small decline between 2009 and 2011, followed by an almost continuous recovery until 2019. However, 2019 was a turning point since the coverage of GPs has declined steadily since then. Table A1 and Fig. A1, in the appendix, provide further details on differences across local groups of primary care practices.

GP coverage rate depends on several factors. Two key variables of interest include the number of enrolled patients in primary care units (demand-side), and the number of GPs measured in full-time equivalent (supply-side). Figures A2 and A3, available in the appendix, display the evolution of those two variables. Between 2009 and 2023, the number of enrolled patients in primary care units decreased by 7%, while the number of GPs FTE also decreased by 5%.

**Fig. 2:**
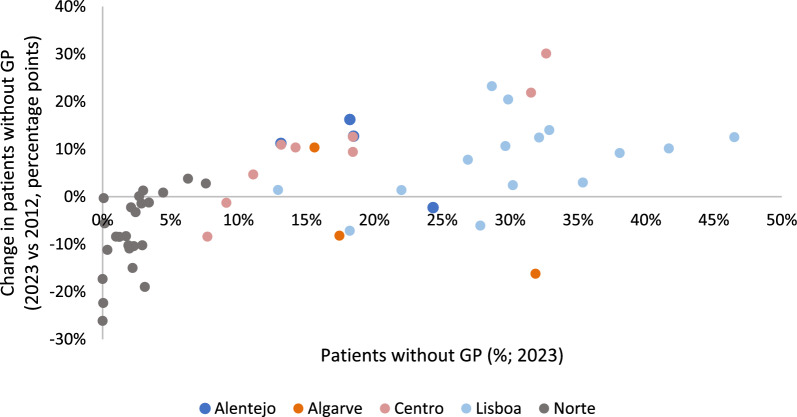
Relation between the change in patients without GP (2023 vs 2012) and the proportion of individuals without GP in 2023 (% of enrolled patients by groups of primary care units)

The proportion of patients without GP is dynamic and heterogeneous across time and regions. Figure 2 displays the relationship between the change in patients without GP (2023 vs 2012) and the proportion of patients without GP in 2023. We are considering long-run trends over this period, even though the effects are not linear over time, as discussed below.

In the *Lisboa* region, for instance, there is significant variability in the proportion of patients without GP, even though most groups of primary care practices (ACES) in this region saw an increase in the proportion of patients without GP. The same does not happen for practices in the *Norte* region, were most ACES displayed higher coverage rates and large improvements in coverage.

Other factor that may play a key role is related with the average number of patients assigned to a GP per doctor, referred to as the patient-to-GP ratio. This does not represent the ratio of enrolled patients per GP, but instead the ratio of enrolled patients with GP per GP. Thus, this ratio reflects the average size of a GP’s list, and can be interpreted as a productivity variable for physicians.

The correlation between this variable and other healthcare and demographic variables is investigated in Table A2, available in the appendix, although no strong correlation was identified. Results suggest a negligible positive correlation with the proportion of patients without an assigned GP. Moreover, a moderate negative correlation exists with both the proportion of elderly individuals and diabetes prevalence. Additionally, a weak negative correlation is observed with population density. This issue was further investigated through scatter plots (appendix A4 to A6).

### Decomposition

**Fig. 3:**
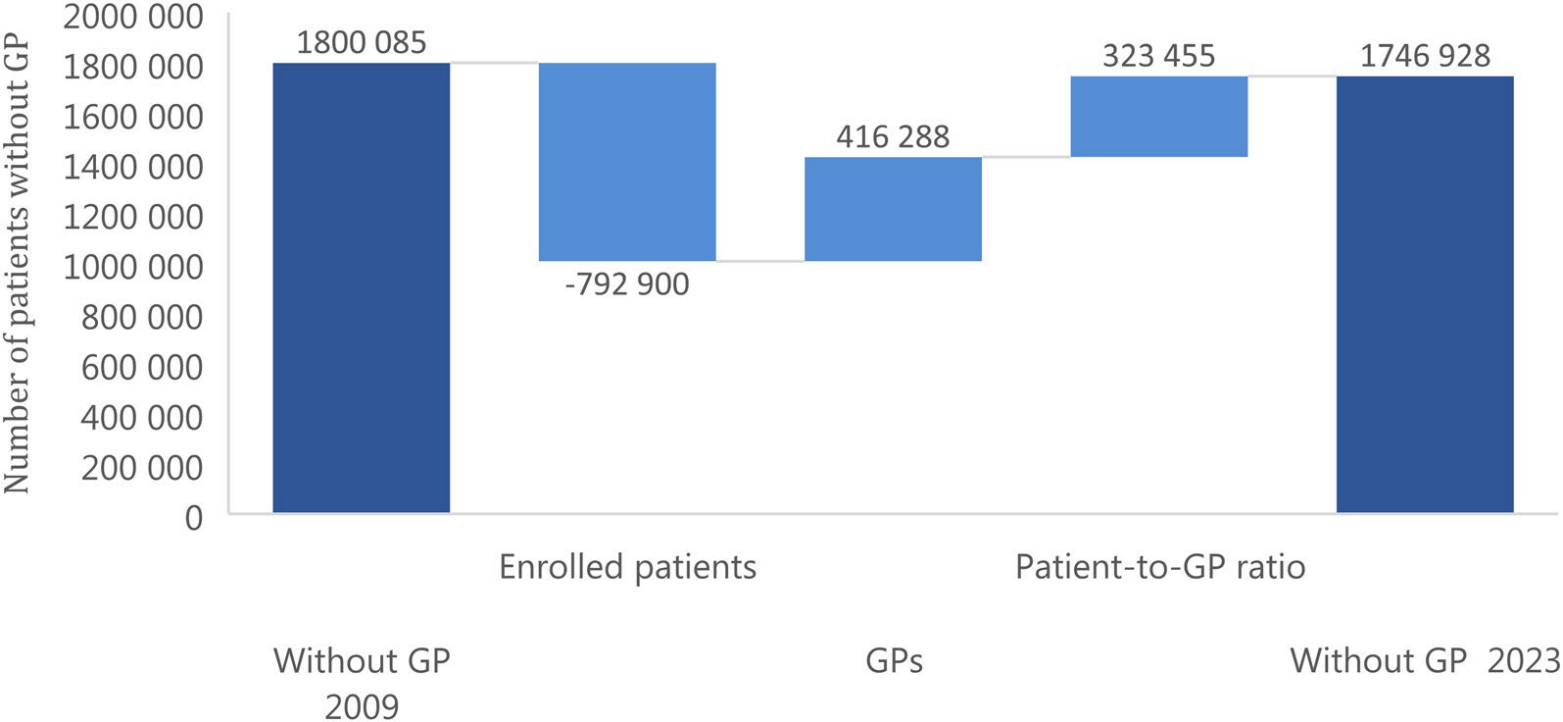
Contribution of each effect (enrolled patients, GP, patient-to-GP ratio) to the change in the number of patients without GP between 2009 and 2023

As explained before, one can decompose the change in the number of patients without GP in these three effects (plus a crossed effect, allocated to the list dimension). The following figure describes the role of each factor (Fig. 3).

Over time, the reduction in the number of enrolled patients in primary care units—capturing a demand-side effect—led to a reduction in the number of patients without GP. This contributed to a reduction of approximately 800 thousand patients without GP. If everything else would remain constant, the number of patients without GP would be reduced by 44% due to the reduction in the number of enrolled patients.

However, during the same period, the number of full-time equivalent GPs has also decreased from 5 650 FTE to 5 395 FTE in 2023 (a 5% reduction)—representing a supply-side effect. By itself, such lack of GPs contributes to an increase in over 400 thousand patients without GP. This corresponds to a 23% increase in the number of patients without GP.

Surprisingly, and often ignored in public discussion, is the impact of the patient-to-GP ratio, which captures a productivity dimension. During this period, the average number of patients assigned to a GP per GP has declined. This implies that more doctors would be required to take care of the same number of patients. In fact, we estimate that the reduction in the patient-to-GP ratio has contributed to an increase of over 300 thousand patients without GP (18%).

This effect may be explained by different factors. However, since our estimates are based on aggregate data, it is not possible to disentangle the relative importance of each potential mechanism. In fact, the reduction in the patient-to-GP ratio may be linked either with an actual reduction of the number of patients in physicians’ lists (for example due to the increased complexity of patients [32]), or by an exit of physicians with larger lists—which reduces the average list size. Further details on these mechanisms are provided in the discussion section.

Overall, even though the reduction in the number of enrolled patients contributes to alleviate the pressure on the coverage rate, the reduction in the number of GPs and in the patient-to-GP ratio had a reverse effect on GP coverage.

**Fig. 4:**
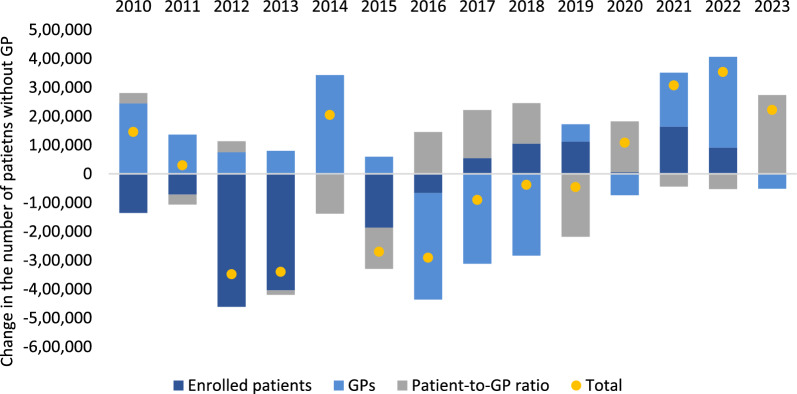
Yearly decomposition of the change in the number of patients without GP (2009–2023). “Total” represents the absolute early changes on the number of patients without GP

These aggregate effects are estimated over a relatively long period of time. However, these three effects may have different contributions throughout time as presented in Fig. 4. Up until 2016, the reduction in the number of enrolled patients (dark blue bar) contributed to an improvement in the coverage rate. To some extent, this may be associated with administrative measures implemented in 2012 and 2013 to remove non-users from primary care. However, since 2017, additional patients have been enrolled in the system. This had the opposite effect, negatively impacting the coverage rate.

Up until 2014, there was a reduction in the number of GPs (light blue bar) which led to a reduction of coverage rates. This situation was partially reversed in the following years until 2018. However, in 2019, and particularly in 2021 and 2022, the change in the number of GPs had a large negative effect in the coverage rate.

Finally, since 2016, and except for the 2019, 2021 and 2022, there was a negative effect on the coverage rate due to a deterioration on the patient-to-GP ratio (grey bar).

**Fig. 5:**
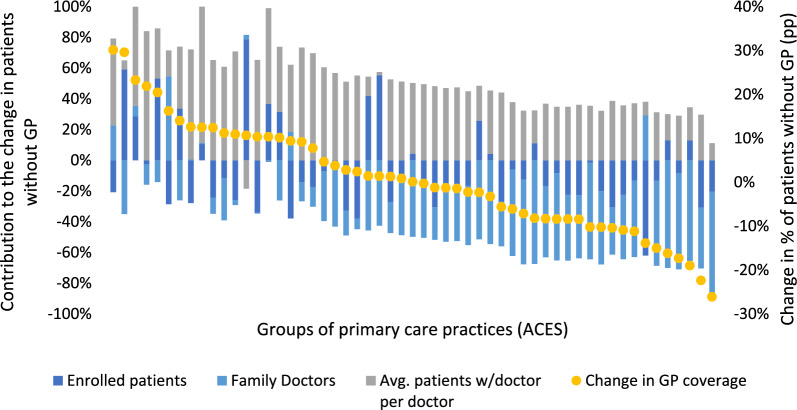
Relative contribution of each effect to the change in the number of patients without GP per group of primary care practice (2012–2023, columns add to 100% (left axis); groups of primary care practices ordered from larger increases in the % of patients without GP to larger reductions in the % of patients without GP (right axis))

There is also heterogeneity across groups of primary care practices (ACES), as displayed by Fig. 5. This plot represents, for each ACES, the relative contribution of each of the three effects to the change in the number of patients without GP.

One can observe some trends which are common to most—but not all—groups. First, most ACES saw a reduction in the number of enrolled patients. Everything else constant, this demand-side effect contributes to reducing the number of patients without GP. Only 15 ACES (27%) saw an increase in the number of enrolled patients.

Second, most ACES were able to hire more doctors, measured in full-time equivalents (supply-side effect). Everything else constant, hiring additional doctors contributes to alleviate the number of patients without GP. Only 6 ACES (11%) saw a negative impact from this supply-side effect.

Third, there was a reduction in patient-to-GP ratio across most ACES. Everything else constant, this reduction contributes to increase the number of patients without GP. Only 1 ACES (2%) saw a positive contribution from this productivity effect.

It is also interesting to note that the negative contribution from the change in patient-to-GP ratio was smaller in ACES that were able to achieve sizeable reductions in the proportion of patients without GP (closer to the right-hand side of the plot). These practices were able to attract and recruit new doctors, compared with practices that saw sizeable increases in patients without GP.

## Discussion

From 2009 to 2023, there has been a slight decline in the number of patients without a GP, decreasing from 1.8 million to 1.7 million patients. However, within this period, significant variations occurred, with a pronounced increase of patients without GP in recent years. The variation in GP coverage may be attributed to three different factors, as highlighted by the previous section.

Between 2009 and 2023, there was an overall decrease in the number of enrollees in primary care (demand-side effect)—a reduction of approximately 793 thousand patients (-7%). During the same period, there was also a net outflow of GPs. These reductions in the number of doctors contributed to an increase in the number of patients without GP by around 416 thousand patients (supply-side effect). Additionally, there was a progressive reduction in the overall number of patients with a GP per physician (patient-to-GP ratio, capturing a productivity effect). This effect was sufficient to counteract the decline in the number of enrolled patients.

### Enrolled patients in primary care (demand-side effect)

The initial decline in the number of patients enrolled in primary care, followed by a subsequent increase from 2016 onward, can be attributed to various factors. Demographic shifts and migratory patterns, marked by low birth rates [33], increased mortality, and stagnant immigration, suggest a foreseeable reduction in enrolled patients in the medium term. Conversely, changes in socio-economic conditions may prompt residents to seek NHS care due to deteriorating health and economic circumstances that hinder the affordability of private healthcare.

Overall, there was a 2.1% population decline from the 2011 to 2021 census. Interestingly, the pandemic may have prompted individuals not previously registered in primary care to seek enrollment, driven by the need for COVID-19 vaccination. Additionally, the recent increase in migration and telecommuting may further contribute to an increase in the number of users.

Moreover, administrative measures implemented in the context of the financial crisis removed non-users from primary care. In fact, between 2012 and 2013, the number of enrolled patients in primary care was reduced by over 800 thousand, largely due to such reset on administrative records. The fact that the number of enrolled patients in primary care is still above the overall Portuguese population, suggests the need to further improve the accuracy of existing official registries.

### Number of full-time equivalent GPs in primary care (supply-side effect)

The mathematical decomposition reveals that the hiring of GPs, in 2015 to 2018, 2020, and 2023, significantly contributed to reducing the number of patients without a GP. However, these new hires were insufficient to assign a doctor to all patients. This may be related to where new hires are being placed. If these were concentrated in practices with a significant shortage of doctors, such as those with small teams at risk of closure due to a lack of professionals, then these hirings, while important for meeting the respective population's needs, did little to resolve the "chronic" lists of patients without a GP in large practices serving vast populations in suburban regions, for example.

Historically spatial maldistribution persists in Portugal despite increased physician supply [34]. The maps presented in the appendix (Fig. A1) highlight the dynamic evolution in the ratio of patients lacking a designated GP, within each local group of practices (ACES). This ratio ranged between 0 and 46% across different local areas. Studying the patterns within each local group reveals geographical disparities, despite temporal fluctuations. Some ACES consistently grapple with a higher prevalence of patients lacking access to a GP relative to their population size, compared to others. However, our findings indicate only a weak correlation (correlation coefficient = 0.05) between the ratio of patients without a GP and the number of doctors per district. This suggests that the maldistribution of physicians may not be the primary factor contributing to the observed disparities between regions.

Additionally, it is crucial to understand which practices attracted applications. A well-known problem in many countries, including Portugal, is the retention of doctors in remote or underserved areas. Isolated strategies have been implemented, including incentives for medical students to choose family medicine or work in underserved regions, and recruiting foreign physicians through bilateral agreements [35]. The incentive scheme introduced in 2015 to attract physicians to underserved areas [16] aimed to enhance practice attractiveness. However, its effectiveness in retaining professionals remains unknown. Potential barriers to hiring doctors include the decreasing real remuneration for physicians in Portugal, with a 21% decrease from 2010 to 2021 [23, 36], leading many professionals to seek better-paid work opportunities abroad [37]. Some authors have also pointed to the insufficient number of places in general and family medicine specialty training. In 2014 around 30% of all vacancies in specialized training for doctors are in family medicine [38].

### Patient-to-GP ratio (productivity effect)

An intriguing finding from this study is associated with the effect of the average number of patients with an assigned GP per GP. The fluctuation in this patient-to-GP ratio, especially in 2016 to 2018, 2020, and 2023, indicates that smaller patient lists might have contributed to the increase in the number of patients without a doctor.

The size of GP patient lists has been the subject of much debate in Portugal. In 2007 the concept of weighted units was introduced, where younger and elder age groups weigh more at the GP list since they represent an increased workload. A weighting factor 1.5 was applied for children aged 0 to 6 years, 2 for adults between 65 and 74 years and a weighting factor 2.5 for adults aged 75 and over years old. In 2007, the minimum list size by each GP was defined as 1,917 weighted units, which corresponded to an average of 1,550 patients in the list [39]. The State Budget Law for 2021 [40] limited patient lists to a maximum of 1,917 patient-weighted units for new GP. While this measure creates the right incentives for providing better care, it does not contribute to reducing the number of patients without a GP. The average panel size lies around 1 700 patients per GP, but many extreme situations exist where GP lists are composed of less than 1 000 patients and more than 2 000 patients [41]. This panel size is in line with other countries rules (Denmark 1 600, England between 1 807 and 2 686, Norway up to 2 500 patients).

On the one hand, migratory movements and demographic changes suggest an increase on doctors' lists of the elderly population and migrant pregnant women [41]. Since risk adjustment was based solely on the age of the population on their lists, the surge in the population with greater care and assistance needs, such as the elderly, is reflected in the number of patients weighted units. In fact, the number of weighted units per enrolled patient has been increasing over time. Our data show that, in 2009, there were 1.24 weighted units per enrolled patient, which increased by 5% to 1.32 in 2023. This implies that additional GPs—at least 200—are required to cover the same overall number of patients, just because of demographic changes.

On the other hand, shifting distribution of doctors between organizational models may also play a role. Doctors in Personalized Health Care Units (PHCU) typically have lists with fewer patients but follow more patients without a doctor, while those in Family Health Units (FHU) manage larger lists but provide limited services to the population without a doctor. Thus, if the recent hiring of GPs has placed more professionals in PHCU at the expense of FHU, this will mathematically reduce the average number of patients per doctor. Similarly, exit of physicians with larger lists may also contribute to reduce the patient-to-GP ratio. This is particularly relevant if older physicians that retire have larger patient lists than younger new hires.

Moreover, it is essential to ascertain whether the geographic location chosen by doctors for practice imposes limitations on expanding care to more patients. If doctors are hired in sparsely populated areas, the number of patients to fill their lists is limited.

Overall, this research suggests that the patient-to-GP ratio is a key variable, often ignored in public discussion which tends to focus on demand (enrolled patients) and supply factors (number of GPs). In the context of the workforce crisis in primary care faced by numerous countries [26], it is important that policy-makers consider the impact of such factor on attaining universal coverage of primary health care. Policies to improve the way such ratio is defined—and exploring alternatives implemented by different countries—may contribute to mitigate the negative effects from the current GP crisis in Europe.

### Limitations and further research

While this descriptive study sheds light on the challenges of the expansion of primary care coverage, there are certain limitations that warrant consideration. The level of detail and causal analysis are limited considering the aggregate nature of the data used in this paper. In fact, the unit of observation is each group of primary care practices (ACES), while differences may also occur between GPs working in the same ACES. Further studies could delve into the dynamics of team composition within practices. Another aspect of the current organizational changes to the FHU model coupled with other demographic changes is their impact on the patient list composition.

## Conclusion

This study provides a comprehensive analysis of GP coverage dynamics in Portugal. We decompose the variation in the number of patients without a GP in three factors.

Between 2009 and 2023, there was an overall decrease in the number of enrollees in primary care (demand-side effect). During the same period, there was also a net outflow of GP, measured in full-time equivalents (supply-side effect). Additionally, there was a progressive reduction in the patient-to-GP ratio (productivity effect). This effect of a gradual reduction in the number of patients in each doctor’s list was sufficient to counteract the decline in the number of enrolled patients, contributing to increasing the number of patients without GP in recent years.

The study highlights the complexity of managing human resources allocation to achieve optimal health care coverage. A proper understanding of the challenges regarding GP coverage is critical to enhance the efficiency of primary care services. In particular, the study emphasizes the importance of strategic planning, efficient recruitment practices, and ongoing evaluation of the effectiveness of initiatives designed to improve GP coverage.

## Figures and Tables

**Fig. 6:**
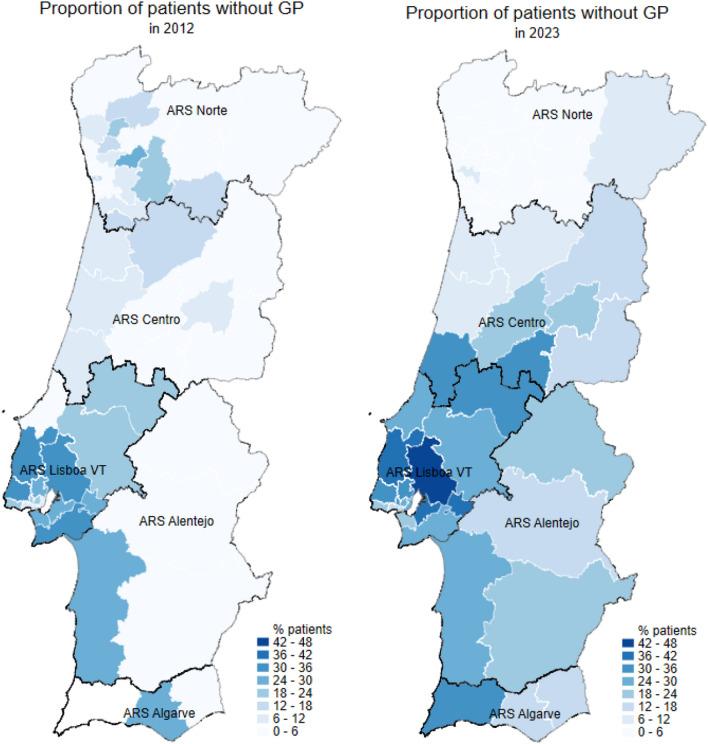
Distribution of patients without GP across regions (% of enrolled patients in primary care)

**Fig. 7:**
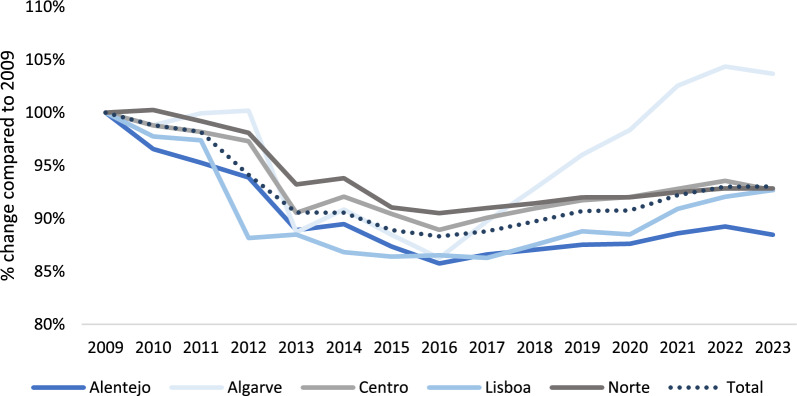
Change in the volume of enrolled patients in primary care units (2009 = 100; 2009—2023)

**Fig. 8:**
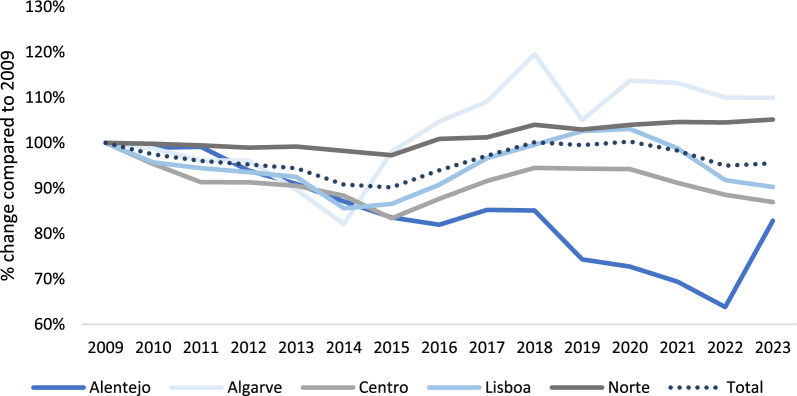
Change in the number of GPs in primary care units (2009 = 100; 2009—2023)

**Fig. 9:**
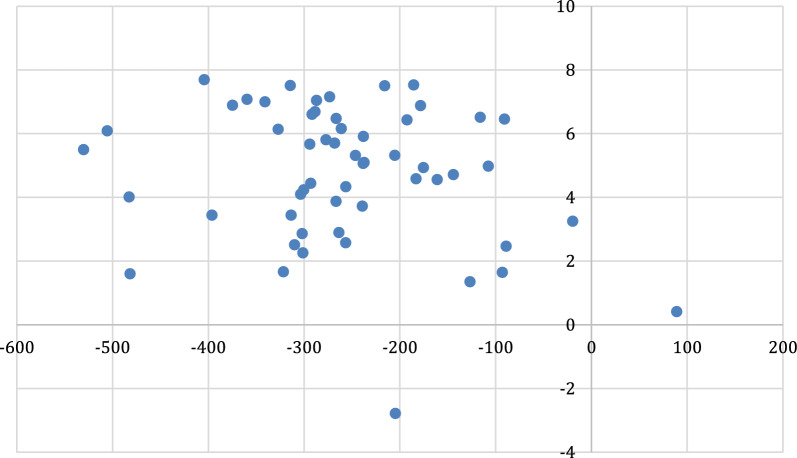
Relationship between the variation in the average number of patients per GP in each ACES (horizontal axis) with changes in the proportion of elderly patients (vertical axis) (2012 versus 2023; %)

**Fig. 10:**
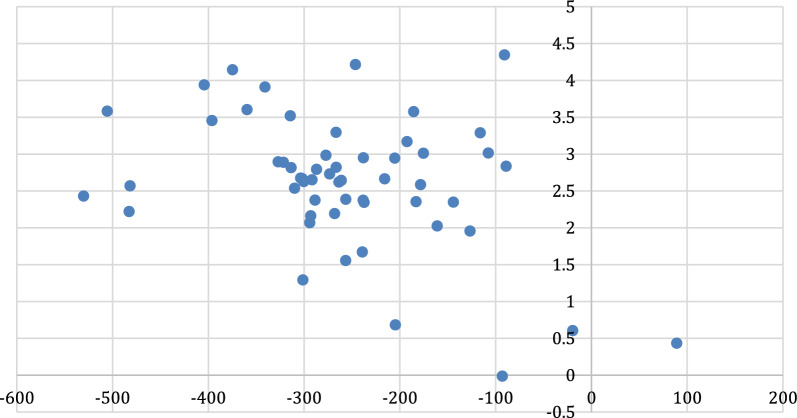
Relationship between the variation in the average number of patients per GP in each ACES (horizontal axis) with changes in the prevalence of diabetic patients (vertical axis) (2012 versus 2023; %)

**Fig. 11:**
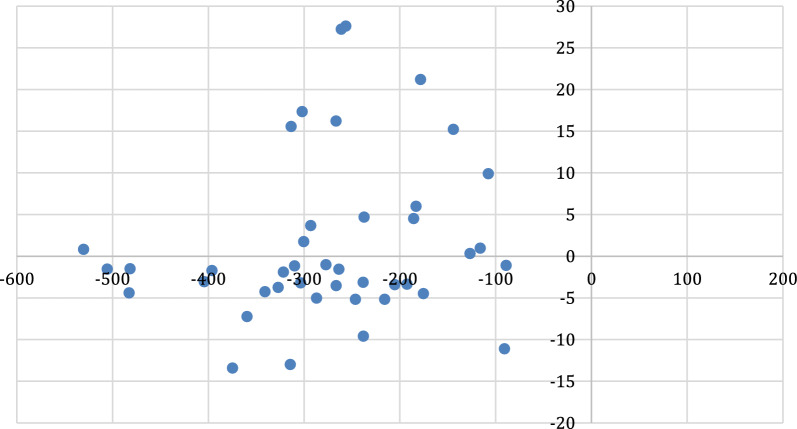
Relationship between the variation in the average number of patients per GP in each ACES (horizontal axis) with population density (vertical axis) (2012 versus 2023; %)

## Data Availability

Data analyzed in this study are available in the Portal da Transparência SNS, available at https://www.sns.gov.pt/transparencia/, in the BI-CSP dashboards, available at https://bicsp.min-saude.pt/pt/biufs/Paginas/default.aspx, and in the ACSS Report on Access to Health Care 2022, available at https://www.acss.min-saude.pt/wp-content/uploads/2022/09/Relat%C3%B3rio-de-Acesso-2021.pdf

## Appendix

See Tables 1 and 2 and Figs. 6, 7, 8, 9, 10 and 11 here.

**Table 1:** Patients enrolled in a primary care practice without a GP assigned per group of primary care practices (ACES; n = 55) (% of enrolled patients in primary care units; 2013–2023)

Group of primary care practices (ACES)	2013	2014	2015	2016	2017	2018	2019	2020	2021	2022	2023
Alentejo Central	1.8	3.5	0.9	1.2	1.8	1.0	1.9	3.8	4.8	13.9	13.1
Alentejo Litoral	24.6	31.5	26.9	14.3	10.9	11.1	13.0	16.6	12.5	16.9	24.3
Algarve Barlavento	51.1	55.7	36.9	33.6	28.8	27.0	21.5	22.7	27.5	28.1	31.9
Algarve Central	23.9	30.2	21.8	12.4	9.2	5.6	10.1	9.2	11.1	15.6	17.4
Algarve Sotavento	6.2	6.1	3.9	2.2	3.4	3.0	3.5	4.1	6.6	13.9	15.6
Almada/Seixal	20.1	24.1	18.2	17.0	11.0	11.5	9.2	10.7	14.7	14.5	18.2
Alto Ave	3.8	3.4	1.4	1.5	1.2	0.1	0.7	1.6	3.0	2.3	4.5
Alto Minho	2.1	3.0	1.5	1.5	1.9	1.0	0.2	1.2	0.3	3.7	3.0
Alto Tâmega e Barroso	2.3	3.9	6.2	3.6	4.1	1.0	2.2	4.1	3.9	0.8	3.4
Amadora	20.8	31.2	28.9	21.5	26.2	29.5	28.9	27.7	27.3	34.1	32.9
Arco Ribeirinho	23.8	23.5	27.5	28.9	22.1	23.5	19.6	24.5	27.1	36.9	38.1
Arrábida	26.3	31.1	29.0	26.8	27.5	26.2	24.9	21.8	23.1	25.5	27.8
Ave / Famalicão	6.8	5.8	4.5	5.2	1.0	1.6	0.4	3.0	0.3	6.6	1.9
Aveiro Norte	12.9	6.2	3.7	0.2	0.1	0.1	0.1	1.6	0.1	0.0	0.0
Baixo Alentejo	4.4	1.6	3.8	1.9	2.7	3.8	4.8	3.9	9.3	17.4	18.2
Baixo Mondego	8.6	8.9	5.3	3.9	3.3	4.7	4.4	5.7	6.4	9.4	9.1
Baixo Tâmega	19.3	24.5	16.6	8.1	3.1	2.3	2.7	2.3	0.5	2.1	0.1
Baixo Vouga	3.3	5.9	5.8	2.3	2.1	3.7	2.9	7.1	6.0	9.0	11.1
Barcelos / Esposende	4.4	5.5	0.8	1.2	3.9	0.4	3.7	6.0	2.3	3.1	1.0
Beira Interior Sul	2.0	3.6	2.9	7.6	7.5	3.8	2.9	6.4	12.3	16.0	13.2
Braga	14.0	3.3	2.6	0.0	3.1	3.0	0.9	2.3	2.1	2.4	3.1
Cascais	20.4	25.5	24.6	17.3	12.0	11.4	12.4	11.5	18.6	20.8	22.0
Cova da Beira	10.3	4.2	6.8	4.3	2.7	4.5	1.5	0.3	3.0	9.8	18.4
Dão Lafões	11.2	10.7	7.0	1.7	4.2	2.3	2.5	2.5	4.9	5.8	7.7
Douro Sul	10.3	10.3	15.7	7.2	3.4	2.2	0.7	2.6	0.3	2.2	2.3
Espinho / Gaia	1.5	3.1	0.5	2.5	2.7	2.7	3.1	2.4	7.2	2.1	2.9
Estuário do Tejo	21.9	34.0	31.0	28.9	29.3	19.2	14.6	20.2	27.4	39.4	46.5
Feira e Arouca	7.8	8.2	2.5	1.5	1.7	0.7	0.9	6.0	3.3	4.2	1.2
Gaia	5.3	5.8	3.0	0.9	2.4	3.7	5.4	6.1	1.3	1.0	2.4
Gerês / Cabreira	7.4	6.7	7.0	5.2	7.4	3.5	3.4	1.2	1.5	1.4	2.0
Gondomar	1.0	2.9	0.6	0.3	0.7	1.2	0.1	4.8	2.3	1.2	0.1
Guarda	4.5	7.2	7.7	6.0	3.7	6.6	4.2	3.4	9.6	12.7	14.2
Lezíria	15.0	24.5	22.0	12.7	12.2	13.9	9.9	10.4	14.3	23.7	26.9
Lisboa Central	6.3	13.9	17.7	15.3	15.2	14.4	12.6	13.8	18.9	27.5	29.9
Lisboa Norte	18.4	18.3	16.4	13.2	10.6	9.8	8.7	10.0	13.9	23.8	29.7
Lisboa Ocidental e Oeiras	10.4	13.2	14.3	9.8	5.4	7.8	10.0	8.9	15.7	18.2	12.9
Loures / Odivelas	19.1	20.4	20.1	14.2	12.4	14.8	11.7	12.0	20.3	25.9	30.2
Maia / Valongo	1.8	3.8	2.0	2.4	3.4	2.2	0.6	3.0	2.6	2.3	6.3
Marão e Douro Norte	3.7	3.5	4.4	2.8	0.4	1.8	3.1	1.7	5.6	3.3	0.2
Matosinhos	3.8	3.4	3.3	2.5	2.8	2.5	1.6	0.7	3.5	2.6	2.1
Médio Tejo	17.5	18.4	16.1	12.5	11.3	6.8	9.3	8.0	18.0	24.6	32.1
Nordeste	3.3	5.0	4.8	2.4	3.2	1.2	1.3	4.2	4.2	5.4	7.6
Oeste Norte	10.2	13.2	12.1	11.8	9.6	16.0	8.0	6.1	21.1	25.2	28.7
Oeste Sul	26.6	32.8	26.9	24.7	14.6	14.2	14.5	9.2	20.9	35.0	41.7
Pinhal Interior Norte	2.8	5.8	10.6	4.0	2.8	4.9	4.9	3.4	6.3	9.1	18.4
Pinhal Interior Sul	1.9	4.4	2.0	1.1	0.7	0.6	1.9	11.9	14.5	14.4	32.7
Pinhal Litoral	16.4	16.7	11.0	4.8	5.0	1.9	2.0	4.3	9.1	14.0	31.6
Porto Ocidental	6.3	10.5	5.7	1.3	3.3	1.6	3.9	6.2	4.0	2.9	2.9
Porto Oriental	7.2	5.4	4.6	1.3	2.0	2.1	3.4	10.4	8.5	5.5	2.2
Póvoa do Varzim / Vila do Conde	1.8	5.1	1.3	2.3	2.4	0.0	0.0	2.4	0.4	3.3	2.7
Santo Tirso / Trofa	1.6	2.2	3.2	1.8	1.4	0.2	0.5	0.9	3.2	0.7	0.4
São Mamede	7.7	11.9	7.9	5.0	4.1	5.9	7.9	6.7	15.4	17.3	18.5
Sintra	29.6	34.7	27.1	24.3	23.8	22.1	23.8	26.2	30.8	33.1	35.4
Vale do Sousa Norte	17.9	10.3	1.9	1.1	4.1	5.4	1.2	1.2	6.6	0.0	0.0
Vale do Sousa Sul	6.0	4.5	3.7	1.5	5.3	1.0	0.0	3.2	2.1	6.0	1.7

**Table 2:** Correlation matrix between the average number of patients per doctor and other healthcare and demographic variables (2012–2023)

	*(1)*	*(2)*	*(3)*	*(4)*
(1) Average number of patients per doctor	1			
(2) % without doctor	0.018412196	1		
(3) Proportion of elderly	− 0.298473198	− 0.21185	1	
(4) Diabetes prevalence	− 0.109616155	− 0.47161	0.600751	1
(5) Population density	− 0.022285867	0.127399	− 0.17077	− 0.29764
